# The impact of active research involvement of young children in the design of a new stereotest

**DOI:** 10.1186/s40900-020-00194-6

**Published:** 2020-06-06

**Authors:** Therese Casanova, Carla Black, Sheima Rafiq, Jessica Hugill-Jones, Jenny C. A. Read, Kathleen Vancleef

**Affiliations:** 1 Institute of Neuroscience, Newcastle University, Henry Wellcome Building, Framlington Place, Newcastle-upon-Tyne, NE2 4HH UK; 2 Present address: NHS Business Services Authority, Stella House, Goldcrest Way, Newcastle, NE5 8NY UK; 3 Faculty of Medical Sciences, Professional Services, Newcastle University, Framlington Place, Newcastle-upon-Tyne, NE2 4HH UK; 4 Present address: Children’s Acute and Ongoing Needs Service, Salford Royal NHS Foundation Trust, Nye Bevan House, Maclure Rd, Rochdale, OL11 1DR UK; 5 Present address: York Trials Unit, Department of Health Sciences, University of York, York, YO10 5DD UK; 6 Present address: Department of Experimental Psychology, University of Oxford, Anna Watts Building, Radcliffe Observatory Quarter, Woodstock Road, Oxford, OX2 6GG UK

**Keywords:** Patient and public involvement, Stereotest, Children, Test development, PPI, Med tech, Stereopsis, Engagement, Co-production

## Abstract

**Background:**

Although considered important, the direct involvement of young children in research design is scarce and to our knowledge its impact has never been measured. We aim to demonstrate impact of young children’s involvement in improving the understanding of a new 3D eye test or stereotest.

**Methods:**

After a pre-measure of understanding was taken, we explored issues with the test instructions in patient and public involvement (PPI) sessions where children acted as advisers in the test design. Feedback was collected via observations, rating scales and verbal comments. An interdisciplinary panel reviewed the feedback, discussed potential changes to the test design, and decided on the implementation. Subsequently, a post-measure of understanding (Study 1–2) and engagement (Study 3) was collected in a pre-post study design. Six hundred fifty children (2–11.8 years old) took part in the pre-measure, 111 children (1–12 years old) in the subsequent PPI sessions, and 52 children (4–6 years old) in the first post-measure. One hundred twenty-two children (1–12 years old) and unrelated adults took then part in a second series of PPI sessions, and 53 people (2–39 years old) in the final post-measure. Adults were involved to obtain verbal descriptions of the target that could be used to explain the task to children.

**Results:**

Following feedback in Study 1, we added a frame cue and included a shuffle animation. This increased the percentage of correct practice trials from 76 to 97% (t (231) = 14.29, *p* < .001), but more encouragements like ‘Keep going!’ were needed (t (64) = 8.25, *p* < .001). After adding a cardboard demo in Study 2, the percentage of correct trials remained stable but the number of additional instructions given decreased (t (103) = 3.72, *p* < .001) as did the number of encouragements (t (103) = 8.32, *p* < .001). Therefore, changes in test design following children’s feedback significantly improved task understanding.

**Conclusions:**

Our study demonstrates measurable impact of involvement of very young children in research design through accessible activities. The changes implemented following their feedback significantly improved the understanding of our test. Our approach can inform researchers on how to involve young children in research design and can contribute to developing guidelines for involvement of young children in research.

## Plain English summary

The United Nation’s convention on the Right of the Child says that children and young people have the right to be involved in decisions that affect their life, to express their views and to have their views listened to. Applying this right to research has lagged behind other areas, but we now see a growing interest and effort to actively involving children and young people in the design of paediatric research. Because involving young children under the age of 6 is challenging, views have most often been gathered from their parents instead. We provide an example of how young children themselves can be involved in the design of a new 3D eye test and we provide evidence for the beneficial impact it can have. At the start of our research, we noticed that many children had difficulties understanding the instructions of our 3D eye test. We explored these issues in patient and public involvement sessions where children acted as advisers. Following their feedback we included animations and a cardboard demo explaining the test. In a new group of children, we tested these changes and found that nearly all children could now understand the instructions. We have shown that it is possible to involve young children in research design and empower them to express their views themselves rather than through their parents. In addition, we have shown that acting on their feedback and suggestions can have a positive impact on the design of eye health care tests.

## Background

The UK National Advisory Group promoting public involvement in health and social care research, INVOLVE, defines Patient and Public Involvement (PPI) as research which is …” carried out ‘with’ or ‘by’ members of the public rather than ‘to’, ‘about’ or ‘for’ them [1]. This does not include people in their role as research subjects or participants in a study, but rather as advisers or collaborators in the research [2]. Involvement of children in research is encouraged in the guiding principles of the United Nation’s convention on the Right of the Child. Article 12 acknowledges that children and young people have the right to be involved in decisions that affect their life, express their views and have their views listened to [3]. Involving children in research from design to dissemination is therefore no longer a preferred approach but a requirement by most funding bodies [4].

Traditionally, children’s perspectives have been filtered through interpretations of parents and carers rather than children being involved themselves with their unique insights into their own reality [5]. Despite the change in policy and vision, examples of involvement of children in research are very limited, especially across the range of healthcare provision [4, 6, 7]. For instance, on 18th March 2020, the INVOLVE evidence library [8] included 516 works of involvement of people in designing research in the health sector, 22 of which relate to children’s research and PPI. Of these 22, only seven describe original research that involves children, and none outline involvement of children under 6 years old. Dunn and colleagues [4] concluded that despite many “efforts to include children’s voices, translation into research and pedagogical practice is still evolving”.

Whilst securing involvement from adults may be more straightforward, involving children in research can be challenging. The difficulty of choosing age-appropriate involvement methods for young children has been seen as a barrier to involvement [4] and good practice methodology on how PPI with children should be achieved is lacking [2]. Some guidelines are available [5, 9] but they only advise on running discussion groups with young people, generally above 12 years old. Crucially, no evidence is available on the *impact* of PPI with young children upon research to drive the development of standards of good practice [6]. In sum, while toolkits and case studies from national bodies provide starting points from which research groups could *promote* PPI practice, a large evidence base containing research that demonstrates *impact* of children’s involvement does not yet exist.

### Aims

The current paper describes how children were involved in research on a new 3D eye test for children with amblyopia or lazy eye. We describe involvement of children in the design of the research, more specifically in improving children’s understanding of ASTEROID, our new 3D eye test, after initial prototype development [10]. The purpose is to highlight the importance and impact of involving young children in the development of medical technology. Evidence for impact of children’s involvement on research is the all-important next step to ensure that Public and Patient Involvement with children becomes a fundamental part of all kinds of research.

## General methods

### The ASTEROID study

The current PPI study is part of the overall ASTEROID study. During the ASTEROID study we experienced issues that motivated us to conducted PPI with young children. We describe the ASTEROID study here to provide the reader with the broader context of the PPI study.

Stereopsis, stereoscopic or simply stereo vision all refer to the perception of depth via binocular vision, or vision using both eyes. Measuring stereopsis is common in children with suspected amblyopia or strabismus. However, current ways of measuring stereopsis can be unengaging and under-sensitive [11]. To address this, a new test, known as “Accurate STEReotest On a mobile Device” or ASTEROID, was developed on a glasses-free 3D tablet (see Study 1 Methods for a detailed description, see also [12]). To engage children and young people, ASTEROID took the form of an animated game. ASTEROID was validated against a gold standard stereotest and normative data were collected. We observed high correlations between ASTEROID and the gold standard stereotest but anecdotal evidence and feedback gathered during data collection highlighted some issues with understanding the task. We observed a high number of children who were not able to resolve the practice trials. In addition, many children seemed to require additional instructions and examples. Key to a good stereotest is ensuring that each participant understood what “seeing the 3D image” would be like to ensure that any failures were genuinely due to problems with stereo vision rather than understanding test instructions. These initial outcomes triggered an iterative PPI approach.

### Design of the studies

**Fig. 1:**
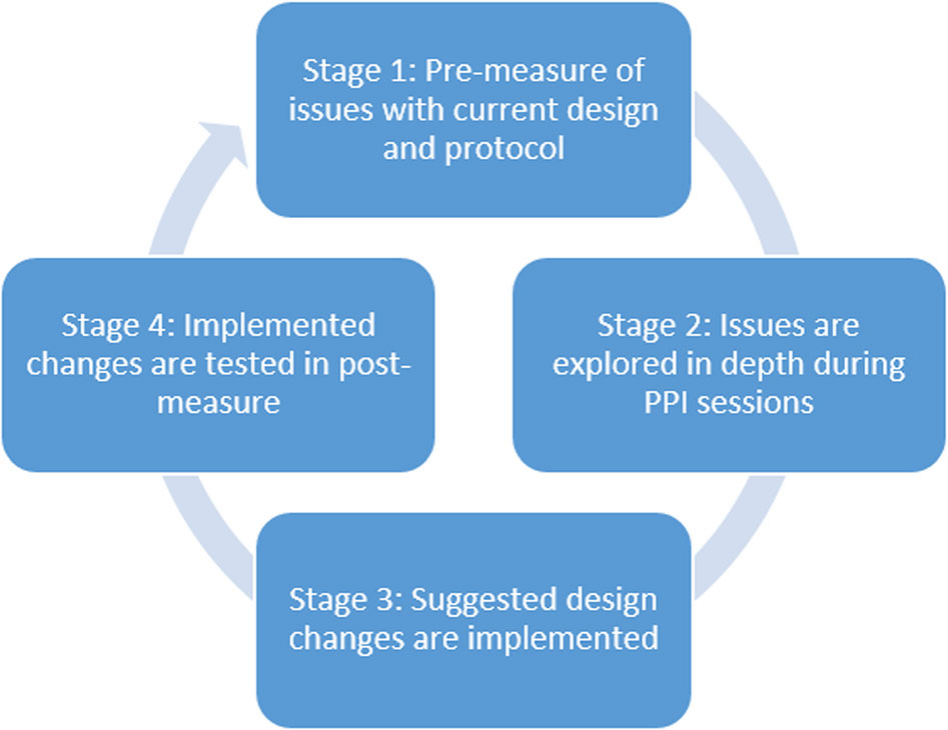
Schematic representation of how PPI was embedded in the development of ASTEROID

An iterative pre-post study design was undertaken to improve ASTEROID (see Fig. 1). Each Study started with a data collection stage in which children completed ASTEROID in the context of our larger validation study (see previous paragraph) and we observed an issue related to poor understanding of ASTEROID (pre-measure). In the second stage, this issue was then explored in depth during PPI with a different group of children or adults until we reached data saturation. By collecting feedback from participants with no previous experience of ASTEROID, we were able to avoid learning effects. PPI outcome triggered changes to ASTEROID (Stage 3). Subsequently, the new version of ASTEROID was evaluated in a separate sample (post-measure, Stage 4). This enabled us to collect quantitative evidence of the impact of our PPI. Children taking part in the pre- and post-measures (Stage 1 and 4) were considered participants and not advisors to the project because the primary outcome was a quantitative measure of understanding and feedback was not explicitly asked for. We did however keep field notes of any spontaneous verbal feedback and observations during Stage 4 which highlighted other issues and subsequently triggered another cycle. In this paper we describe two such cycles that aimed to improve understanding of ASTEROID (Study 1 and 2). Our secondary aim was to monitor how engagement levels changed following improvement of ASTEROID (Study 3). We followed GRIPP reporting guidelines in describing our PPI study [10].

## Study 1: practice trials and trial-to-trial transition

### Stage 1: pre-measure

#### Participants

Six hundred fifty children between 2.1 and 11.8 years old (median age = 6.2, IOQR = 3.1, 318 boys and 332 girls) participated in Stage 1. They completed the ASTEROID stereotest in the context of a larger validation study with other vision tests at school or nursery. All parents received an information leaflet about the study and an opt-out consent form. If requested by the school or nursery an opt-in consent procedure was used. Children were always asked for oral or non-verbal assent at the time of testing. The study protocol was compliant with the Declaration of Helsinki and was approved by the Ethics Committee of the Newcastle University Faculty of Medical Sciences (approval number 01078).

#### Instruments

**Fig. 2:**
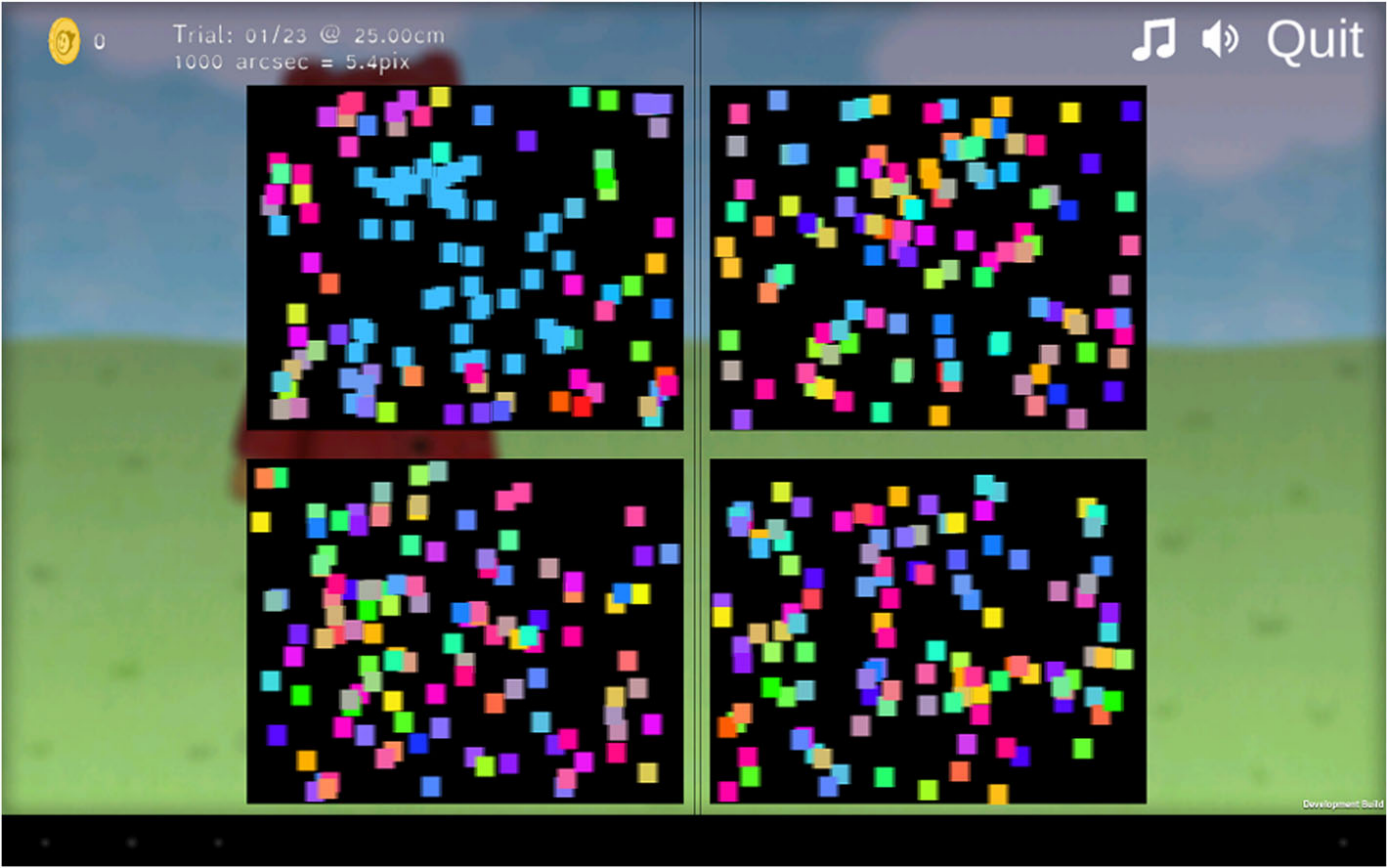
Screenshot of a practice trial of ASTEROID (version 0.933). The tests show four dynamic random-dot stereograms. One of the four stereograms has a square with a different disparity and appears to float above the display. In the practice trials this square also has different coloured dots. The colour cue is removed in the disparity-only trials

ASTEROID (version 0.932 and 0.933) is a stereotest that runs on a 3D tablet computer. The stereotest is embedded in a game designed to keep children engaged and responsive. The test takes the form of four dynamic random-dot stereograms (Fig. 2). A disparate target appears randomly in one of these stereograms. The child is verbally instructed to look at each of the four “squares” individually to see which one sticks out. To ensure that the child understands the task, ASTEROID starts with non-stereo practice trials. In these trials, a colour is added on top of the stereo cue, to make the target clearly visible. If the child solves the practice trials correctly, the colour cue gradually fades away over the next few trials and will eventually disappear, leaving only the stereo cue. Technical details of ASTEROID are described elsewhere [12].

#### Outcome measures

Our primary outcome measures were (1) the proportion of children who solved less than 80% of the practice trials, (2) the proportion of practice trials that were solved correctly by each child, and (3) the number of additional verbal instructions needed for each child. Performance in the practice trials gives us a good indication of the understanding of the task because even children with vision problems like stereoblindness or colourblindness should be able to solve the practice trials correctly. Secondly, observations of children playing ASTEROID were valuable in generating hypotheses for poor understanding of the task that were further explored in Stage 2.

#### Results

39% of the children showed a poor understanding of the ASTEROID task by solving less than 80% of the practice trials correctly. The average proportion of correct practice trials was 0.76 and on average 10 additional prompts were needed per child to explain the task. We particularly observed difficulties in the transition between the practice trials and the stereo trials.

### Stage 2: PPI sessions

#### People involved

One hundred eleven children between 2 and 12 years old (median age = 4, IQR = 3, 53 girls, 55 boys, 3 gender not recorded) were involved in the PPI sessions. They attended local science and history museums in Newcastle-upon-Tyne (United Kingdom) between October 2016 and January 2017 and were invited to join a PPI session for 5–10 min. No ethical approval was required for PPI [13]. Children were thanked for their feedback with a ‘Junior Scientist’ certificate and a sticker.

#### Level and nature of involvement

**Fig. 3:**
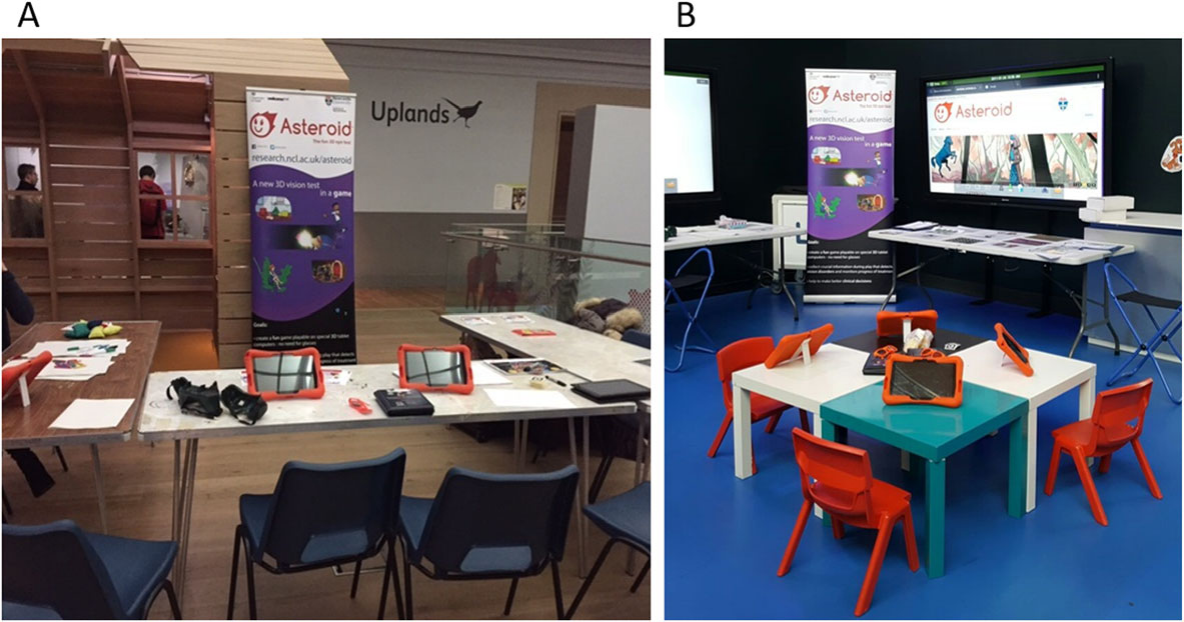
Our set-up for the drop-in PPI activities at Discovery museum (**a**) and Centre for Life (**b**)

We organised three informal drop-in PPI sessions (Table 1). We set-up a stall with engagement activities around vision testing and neuroscience in an area of the museum with high traffic (Fig. 3). The stall contained games including visual distortion goggles, bean-bags and examples of visual illusions. The competitive nature of some of the activities engaged children and their siblings, whilst certificates and other small prizes were available as a reward. Crucially, children were invited to try out ASTEROID (version 0.932 and 0.933, 0.933 has minor technical improvements compared to version 0.932) and comment on any aspect of the design. Sessions were deliberately kept short and informal with a focus on gathering opinions. We tried to avoid giving children the feeling of being tested as research participants. We therefore choose to only collect a minimum of personal details (age and gender) and conduct unstructured interviews with the children.

**Table 1:** Overview of PPI sessions for Study 1

Session number	Date	Venue	Nature of session	Age range	N	ASTEROID version number	Individual feedback provided?^c^	Engagement rating provided?
1	28/10/2016	Discovery Museum^a^	Drop-in session	3–12	26	0.932	No	Yes
2	07/01/2017	Great North Museum: Hancock^a^	Drop-in session	2–11	38	0.933	No	Yes
3	24/01/2017	Centre for Life^b^	Drop-in session	1–6	47	0.933	Yes	Yes

^a^Local museum with free entrance; ^b^Local museum with entrance fee; N = number of people involved; ^c^Individual feedback refers to whether verbal feedback was collected from each child in addition to observations

We asked questions like ‘What are you looking for?’, ‘Can you think out loud while you are playing ASTEROID?’, ‘How come you’re not sure about this one?’, ‘What makes you hesitate?’, ‘What do you find difficult?’, ‘What is unclear to you?'. This information was collected in Session 3 only. In the first two sessions and for younger children, sessions were guided by observations during undirected play that were collected in field notes. The informal unstructured environment also allowed us to observe and question why children would not complete the game but stop prematurely and move on to another activity. This could for instance be related to a lack of engagement of the game but also a lack of understanding. Opinions were gathered by research assistants: CB, TC, JH, and SR. They had experience in engaging with children via their previous roles as an optometrist, orthoptist, a primary school teacher, and a teacher training programme coordinator.

#### Outcome measures

1. Observations of the child’s progress (e.g. focus, hesitation, difficulties etc.) were collated on each trial.
2. Individual verbal feedback from children (session 3 only)

#### Outcomes of PPI

We used principles of thematic analysis to analyse our field notes with observations and verbal feedback given. Observations of some children indicated a lack of understanding of the gameplay. For example, for a 2-year old boy it was noted that he “only played a few trials and showed no understanding of what to do”. A similar observation was made for another 2-year old girl: “Happy to play but didn’t understand”, and for a 1.5 year old girl: “Didn’t understand, mum modelled a lot”. In many children we observed hesitation on how to proceed at the end of the non-stereo practice trials, indicating a gap in understanding on what to do during the non-stereo and stereo stages of the game. In addition, comments made during sessions indicated that the colour cue in combination with the colourful dots in the non-stereo trials primed children to look for a local colour change in a few dots instead of an overall depth change in the stereo trials.

Second, some children had difficulties understanding that on each trial the locations of the target was randomly determined. In subsequent trials, these children would tap all four stereograms in alternation. For instance, if they incorrectly tapped the top left stereogram in one trial, in the next trial they would not revisit this location but instead try the top right location. If the target was not found there, they would move to yet another position in the third trial. This seemed to indicate that children did not understand that the placement was random in each trial, but imagined that the target location remained the same until it was found.

### Stage 3: implementing changes

#### Methods

Feedback from Stage 2 was summarised by research assistants and discussed at a cross-disciplinary meeting attended by vision scientists, computer scientists and game developers. Once consensus was reached, the proposed changes were implemented in a new version of ASTEROID.

#### Results

**Fig. 4:**
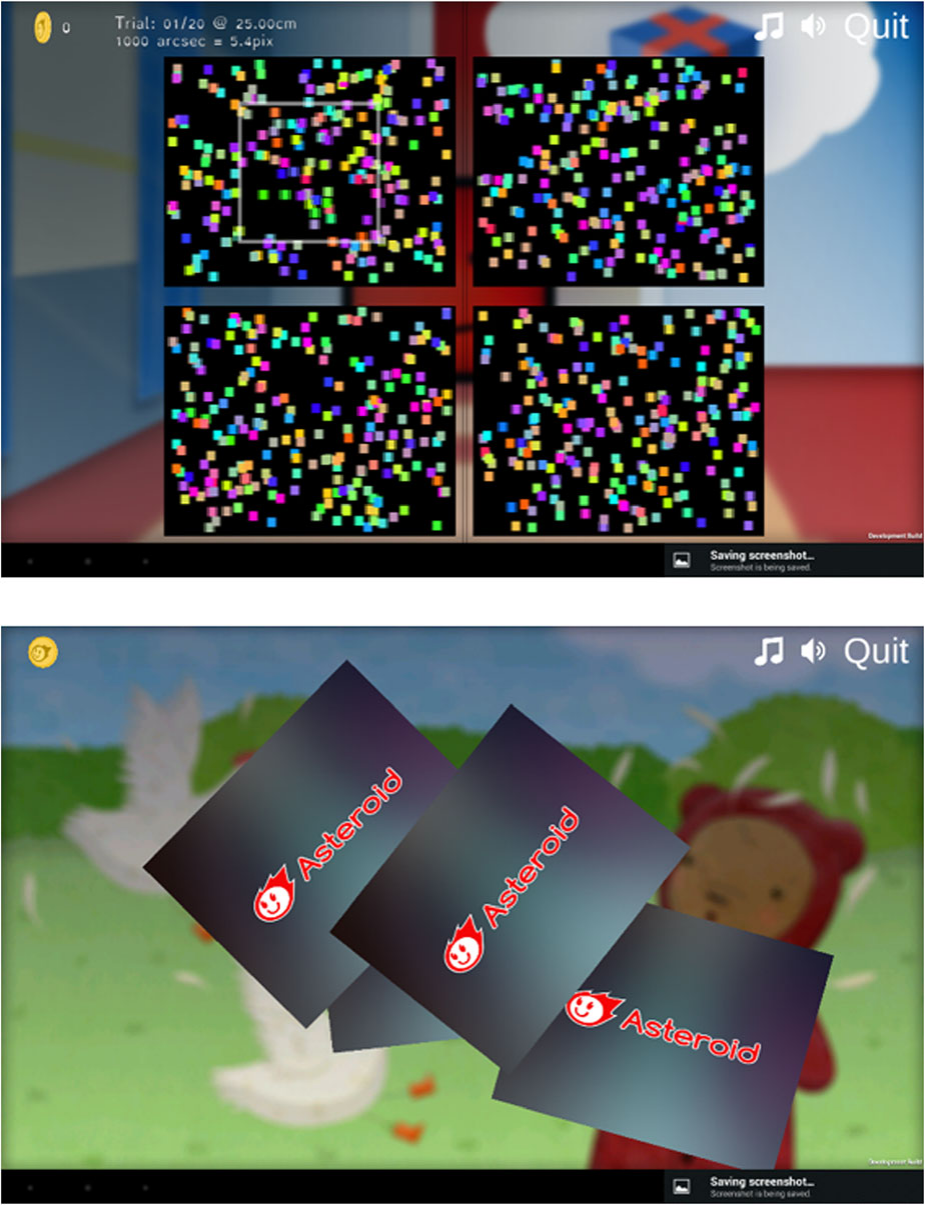
Screenshots (version 0.937) of the changes made after the first Public and Patient Involvement round: a frame cue (top) and a shuffle animation (bottom, see also video at https://youtu.be/w8q-4uejwdk)

To solve the first problem that children focused on the colour change rather than the change in depth, we replaced the colour cue by a frame (Fig. 4). The second problem with understanding the random location of the target in each trial was solved by adding a card shuffle animation. At the end of the trial, the stereograms would flip around, move to the centre of the screen, mix up, move out again to the four corners of the display and flip back face up (see video clip at https://youtu.be/w8q-4uejwdk). The animations mimicked playing cards being shuffled and dealt out. We believed this would explain to young children that in the next trial the target could appear in any of the four locations. Even if a child would have not been exposed to traditional playing cards, they likely have observed the effect of shuffling of jigsaw pieces or cards in children’s bingo games.

### Stage 4: post-measure

#### Participants

Fifty-two children between 4.5 and 6 years old (median age = 5, IQR = 0.4, 27 boys and 25 girls) participated in Stage 4. They completed the ASTEROID stereotest (version 0.938) and other vision tests in the context of a larger validation study.

#### Procedures

Ethical procedures, data collection procedures and outcome measures were the same as in Stage 1.

#### Impact of PPI

**Fig. 5:**
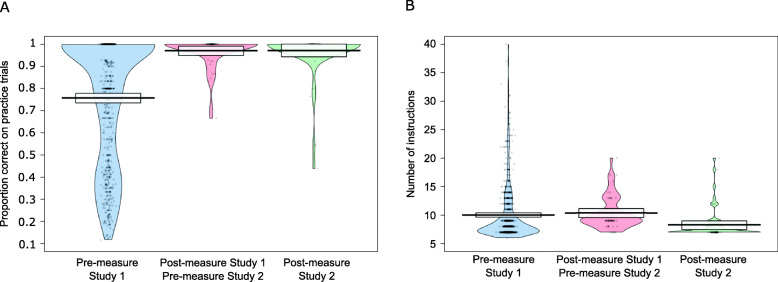
Impact of children’s involvement. Distributions of proportion correctly solved practice trials (**a**) and number of additional instructions (**b**) for the pre- and post-measures of Study 1 and 2. Points represent individual responses, rectangles represent the upper and lower quartile and mean, and blobs represent the spread of the distribution of responses

4% of the children showed a poor understanding of the ASTEROID task (defined as less than 80% correct on the practice trials) after we implemented the shuffle animation and the frame cue in the practice trials, compared to 39% before the changes were made. The proportion of correct practice trials significantly increased from an average of 0.76 in the pre-measure to 0.97 in the post-measure (Welch two-sample t-test: t (231) = 14.29, *p* < .001, d = 1.2, Fig. 5a). However, we observed a similar average number of verbal instructions that needed to be given (mean in both pre and post-measures = 10; Welch two-sample t-test: t (77) = 0.81, *p* = .42, d = 0, Fig. 5b).

## Study 2: instructions

### Stage 1: pre-measure

The post-measure data of Study 1 served as the pre-measure data of Study 2.

### Stage 2: PPI sessions

#### People involved

One hundred twenty-two children and adults were involved in the PPI sessions of Study 2. Children were between 1 and 12 years old (median age = 4, IQR = 4). Thirty-seven of them were girls and 24 of them were boys. Age and gender was not recorded for the 61 adults. The children attended local science and history museums in Newcastle-upon-Tyne (United Kingdom) in February 2017 and were invited to join a PPI session for 5–10 min. Adults were either recruited via our research volunteer pool and registered to attend a session at Newcastle University or they attended a drop-in session at a public venue (Newcastle University Medical School foyer or local museum). Children were thanked for their feedback with a ‘Junior Scientist’ certificate and a sticker.

#### Level and nature of involvement

The public was involved through eight informal PPI sessions (Table 2). All people involved were given the opportunity to try out ASTEROID (version 0.938–0.94, computational processing was sped up in version 0.94) in drop-in sessions similar to those described in Study 1. Children under 12 were asked for individual feedback and engagement ratings. In our previous PPI sessions, children struggled with describing what they were looking for in the stereotrials and with verbalising their though process. To gain more insight in how people work out how to perform the test, we organised PPI sessions with adults. By involving adults, we aimed to obtain a range of verbal descriptions of the target that could be used to explain the task to children.

**Table 2:** Overview of PPI sessions for Study 2

Session number	Date	Venue	Nature of session	Age range	N	ASTEROID version number	Individual feedback provided?^c^	Engagement rating provided?
4	24/02/2017	Discovery Museum^a^	Drop-in session	2–11	27	0.938	Yes	Yes
5	28/02/2017	Centre for Life^b^	Drop-in session	1–4	27	0.938	Yes	Yes
6	05/05/2017	Newcastle University Medical School Foyer	Drop-in session	Adults	14	0.94	Yes	No
7	26/05/2017	Newcastle University, Institute of Neuroscience	Registration required	Adults	8	0.94	Yes	No
8	09/06/2017	Centre for Life^b^	Drop-in session	Adults	9	0.94	Yes	No
9	16/06/2017	Lit&Phil^a^	Drop-in session	Adults	10	0.94	Yes	No
10	23/06/2017	Newcastle University, Institute of Neuroscience	Registration required	Adults	10	0.94	Yes	No
11	30/06/2017	Newcastle University, Institute of Neuroscience	Registration required	Adults	17	0.94	Yes	No

^a^Local museum with free entrance; ^b^Local museum with entrance fee; *N* = number of people involved; ^c^Individual feedback refers to whether verbal feedback was collected from each child in addition to observations

#### Outcome measures

The outcome measures were the same as in Study 1.

#### Outcomes of PPI

Field notes with observations and comments were analysed using principles of thematic analysis. Adults described the target as ‘a square’, ‘centre that sort of sticks out’, ‘popping out and behind’. The outline of the square seems to become more difficult to see at lower disparities when adults described it as ‘a circle appears’, ‘something looks off’, ‘I can see 3D without square’ and even ‘going with the gut’. We used these variety of ways to describe the 3D target to the children but observed that children find it difficult to understand any verbal description of a 3D target. However, once they have seen the target in 3D for the first time, they understand what they need to look out for in the subsequent trials.

### Stage 3: implementing changes

#### Methods

Just as in Study 1, feedback from Stage 2 was discussed at a cross-disciplinary meeting and consensus changes were implemented in a new version of ASTEROID.

#### Results

**Fig. 6:**
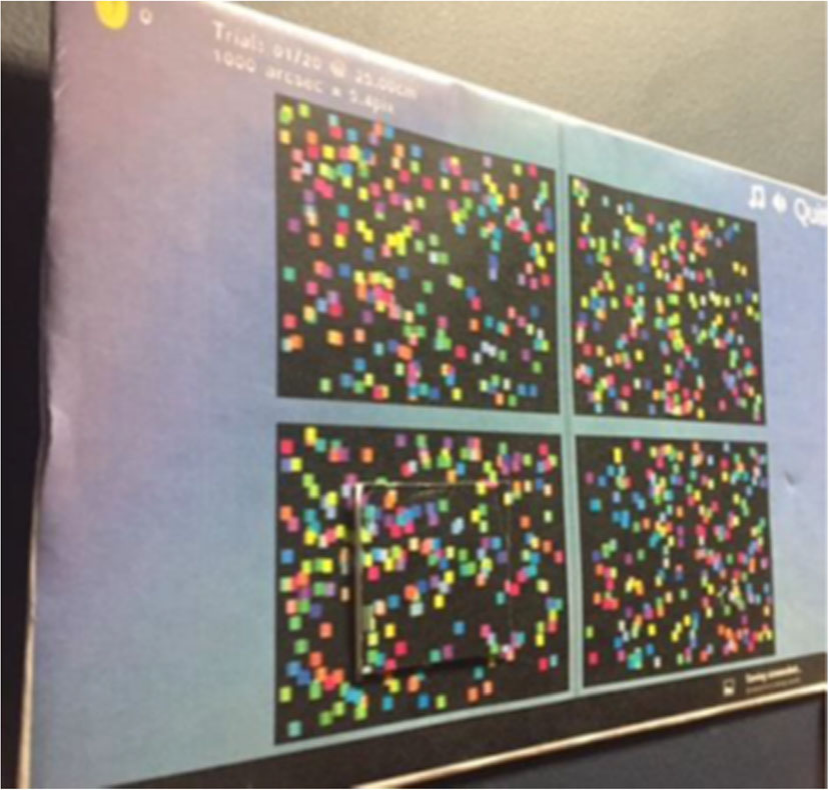
Cardboard demo added after the second Public and Patient Involvement round

We decided to provide the children with a visual and tactile aid to explain them what our 3D target looks like. We therefore made a cardboard demo of an ASTEROID trial. The demo shows a print screen of a trial as the background. In the bottom right location, a square with the same dot pattern as the background was glued on top of the background with a 2 mm cardboard layer in between (Fig. 6).

### Stage 4: post-measure

#### Participants

Fifty-three participants between 2 and 40 years old (median age = 10.8, IQR = 5.1, 17 boys and 36 girls) participated in Stage 4. They completed the ASTEROID stereotest (version 0.940) with additional cardboard instructions in the context of a larger validation study.

#### Procedures

Ethical procedures, data collection procedures and outcome measures were the same as in Study 1 Stage 1.

#### Impact of PPI

6% of the participants showed a poor understanding of the ASTEROID task (defined as less than 80% correct on the practice trials) after we implemented the cardboard instructions compared to 4% without the cardboard instructions. There was no significant increase in average proportion of correct practice trials between pre- and post-measure (mean proportion correct in pre- and post-measure = 0.97; Welch two-sample t-test: t (92) = − 0.02, *p* = .98, d = 0, Fig. 5a). However, we found a significant decrease in the average number of additional verbal instructions given from 10 to 8 (Welch two-sample t-test: t (103) = 3.72, *p* < .001, d = 0.6, Fig. 5b). An age difference between the pre- and post-measure might possibly confound this effect; we therefore reran our analyses including only subjects younger than 9 years old (*n* = 22, median age = 6.3, IQR = 3.4, 8 boys and 14 girls). This indeed resulted in an insignificant difference in the number of additional verbal instructions given (Welch Two sample t-test: t (31) = 0.83, *p* = .41, d = 0.1), however posthoc power calculates indicated a lack of power. A sample of 46 children under 9 would be needed to detect the effect we found in our full sample.

## Study 3: engagement with ASTEROID

Our primary aim was to increase children’s understanding of ASTEROID, however an ASTEROID score can only reflect stereo ability if scores are not inflated by poor motivation. ASTEROID is therefore embedded in a game and different game themes are available to engage children with different interests. We monitored engagement levels during the development of ASTEROID.

### Methods

**Fig. 7:**
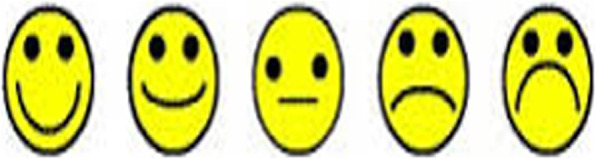
Rating scale for engagement with ASTEROID. The scale was accompanied by the question: Did you enjoy playing the Asteroid game?

Five groups of children and adults were involved in evaluating engagement (Table 3). The groups are described in detail above. Group 4 was a subset of the children and adults involved in Stage 2 of Study 2 (session 4–5). Different outcome measures were collected from different groups (see Table 3). We monitored the number of encouragements given by the researchers during game play, such as ‘Keep going’, ‘Well done’, ‘Just have a go’, etc. In other groups, children rated their level of enjoyment on a smiley face rating scale with five levels (Fig. 7). Our choice of smiley faces was informed by the common use of smiley faces for expressing opinions in primary schools and nurseries as well as in paediatrics and user experience research [14], although the scale was not formally validated for the purpose of our study. Children’s ratings were converted to scores between 1 (saddest face) and 5 (happiest face).

**Table 3:** Groups of people involved in evaluating engagement with ASTEROID

People involved	N	Described in	ASTEROID version	Outcome measure
Group 1	650	Study 1, Stage 1	0.932 or 0.933	Number of encouragements
Group 2	112	Study 1, Stage 2	0.932 or 0.933	Engagement rating
Group 3	52	Study 1, Stage 4	0.938	Number of encouragements
Group 4	53	Study 2, Stage 2	0.937 or 0.938	Engagement rating
Group 5	53	Study 2, Stage 4	0.940 + cardboard demo	Number of encouragements

*N* Number of people involved

### Results

ASTEROID was rated as a fun game by the children with an average rating of 4.8 in Study 1 and 4.7 in Study 2. The implementation of the frame and the shuffle animation did not change the rating (Welch two-sample t-test: t (68) = 0.91, *p* = 0.36, d = 0.2). Although engagement was not the primary aim of the PPI sessions, occasionally positive engagement with ASTEROID was spontaneously noted by the research assistants, for instance ‘Really enjoyed it, laughed at triangle/prize coming up’, ‘Happy to play’, ‘Liked simple [game] but preferred chicken story better’. We found a significant increase of average number of encouragements from 9.6 to 13.8 after we implemented the shuffle animation and the frame cue (Welch two-sample t-test: t (64) = 8.25, *p* < .001, d = 1.1). This indicated that children needed more encouragement and ASTEROID was less engaging after Study 1. However, the average number of encouragements significantly reduced to 8.0 after we added the cardboard demo (Welch two sample t-test: t (103) = 8.32, *p* < .001, d = 1.6).

## Discussion

Our aim was to demonstrate the impact of children’s involvement in the design of a new test that measures 3D vision, ASTEROID. Through two PPI studies, we consulted 233 children and adults. The insights based on their feedback were very valuable for the development team and we made three changes in subsequent versions of ASTEROID: (1) we replaced the colour cue by a frame cue; (2) we added a shuffle animation to explain that the target could appear in any location on the next trial; (3) we made a visual and tactile cardboard demo to explain to the children what target they were looking for in ASTEROID.

The involvement of children and the changes we made to ASTEROID following their feedback had large to very large impact on the level of children’s understanding of ASTEROID (effect sizes 0.6 to 1.2). We noticed a substantial increase from 0.76 to 0.97 in the average proportion of practice trials answered correctly after implementing the frame cue and the shuffle animation. Implementation of the cardboard demo had no effect on the average proportion of practice trials answered correctly. This was likely due to a ceiling effect (97% correct before and after implementation). The impact on the number of additional verbal instructions given to the child during ASTEROID is less straightforward. We observed a slight increase after making the first set of changes, but the number of additional instructions given decreased again after we included the cardboard demo. This seems to indicate that the cardboard demo was able to replace some of the verbal instructions, while the frame cue and shuffle animation were not. Given the nature of the problem that these changes were trying to solve and the type of verbal instructions given, this is not that surprising. Most verbal instructions were variations on what target to look out for: ‘Tap the one that is different’, ‘Which one is sticking out’, ‘Do any look like they are popping out’. The cardboard demo was included because children had difficulties understanding any verbal descriptions of the 3D target. So, including a visual and tactile aid removed the need for additional verbal instructions. The frame cue and the shuffle animation were solving rather different problems, not directly related to the verbal instructions given. Last, we did not see a change in the self-reported engagement levels on our 5-point rating scale with any of the changes we implemented. With an average rating of 4.7 and 4.8 that is likely due to a ceiling effect. This is in agreement with previous research suggesting that children only use the two most ‘happy’ options on a five point smiley face Likert scale [15]. The effect of changes on the number of encouragements that were given during ASTEROID is less consistent. We observed an increase in encouragements after implementation of the frame cue and shuffle animation while we noticed a decrease after implementation of the cardboard demo.

Our study provides a contribution to an evidence pool of involvement of children and young people in research. With typical sample sizes of 5–20 children or young people [16–18], our sample of 233 people (of which 165 were under 12) is a step forward in hearing a wide range of opinions. To our knowledge, our study is the first to involve children as young as 1 year old. 30% of our sample are children of 3 years old or younger. Instead of asking parents’ opinions as is commonly done with children that age [2, 5], we describe methods to successfully gain opinions directly from very young children: using smiley face rating scales for engagement level and using observations and simple questions to investigate understanding of the task. For more in-depth knowledge on the reasoning of solving an ASTEROID trial, we relied on verbal reports from adults unfamiliar with 3D computer tablets. A third strength of our study is the quantitative way in which we measured the impact of our PPI through pre- and post-measures, demonstrating a positive and measurable effect of PPI on research.

The impact of our PPI was possibly limited by the type of people we involved. By running most of our PPI session in public places and through drop-in sessions, we aimed to lower the barriers for involvement. However, most children involved were visiting a local science or history museum with their parents. Museum visitors do not necessarily reflect general demographics. In addition, one of the museum (Centre for Life) has an entrance fee of £11 for adults and £6.50 for children between 5 and 17. This probably caused an underrepresentation of children from lower socio-economic backgrounds. A second limitation of our study is that the median age of participants in our post-measure of Study 2 (median age = 10.8) was higher than in our pre-measure (median age = 5). When subsamples are analysed that are matched for age the effect of the cardboard demo on reducing additional verbal instructions becomes insignificant. This seems to suggest that the effect is mediated by age and is not related to the cardboard demo. However, a posthoc power calculation shows that our subsample of 22 children is too small to pick up a potential effect and a minimum sample size of 46 participants in each group is required. Therefore with the current sample, we cannot answer the question whether the effect should be related to the cardboard demo or to age differences between samples. A larger sample of younger children is needed to confirm this. A final limitation is in the use of adult research assistants. Adult might have interpreted the children’s game play behaviour incorrectly and results might have been biased by adult’s perceptions of children’s behaviour and capabilities. Involving a child researcher might have provided an alternative view. However, our research assistants were trained in and had experience in working with young children (see methods) and our positive results indicate that we have interpreted children’s comments and observations at least partly correctly.

## Conclusions

In conclusion, children’s contributions have measurably impacted on the development of ASTEROID, a new stereotest for children. By increasing accessibility and through creative methods we gathered feedback from 165 children between 1 and 12 years old and 68 older children and adults. The changes implemented following their feedback significantly improved our stereotest. Our approach can be inspirational for future researchers and contribute to an evidence pool of good-practice in involvement of children and young people in research design.

## Data Availability

Data and analysis code are publicly available. Analysis code: https://doi.org/10.6084/m9.figshare.8378582. Datafiles: https://doi.org/10.6084/m9.figshare.8345573 and https://doi.org/10.6084/m9.figshare.8345570.

## Abbreviations

PPI: Patient and public involvement
ASTEROID: Accurate STEReotest On a mobile Device
IQR: Interquartile range
